# Assessment of Patient Attitudes About Mometasone Furoate Nasal Spray: *The Ease-of-Use Patient Survey*

**DOI:** 10.1097/WOX.0b013e3181865f99

**Published:** 2008-09-15

**Authors:** Leonard M Fromer, Gabriel R Ortiz, April M Dowdee

**Affiliations:** 1 University of California, Los Angeles School of Medicine, Los Angeles, CA; 2 Allergy and Asthma Center of El Paso, El Paso, TX; 3 Phadia US Inc, Portage, MI

**Keywords:** ease of use, mometasone furoate, survey, allergic rhinitis

## Abstract

**Background:**

Intranasal corticosteroids (INS) are recommended as first-line therapy for allergic rhinitis. To date, no studies have evaluated the impact of an INS delivery system on patient satisfaction. Unless patients use a medication appropriately and consistently, they will not fully benefit from its therapeutic effects.

**Objective:**

To determine whether the characteristics of the mometasone furoate nasal spray (MFNS) delivery device are an impediment to its use.

**Methods/Database:**

A random sample of preidentified MFNS users was recruited through e-mail (N = 1544). In online interviews, participants were asked about MFNS ease of use and attributes.

**Results:**

Ninety-eight percent of respondents reported that MFNS is easy to use, and 96% said that the applicator is easy to administer. Nearly all elderly users and users with arthritis said that the applicator fits comfortably in the nostril (96% and 97%, respectively) and is appropriately sized (97% and 96%, respectively); 96% of pediatric users agreed that the applicator fits comfortably. All users said the applicator's ease of use and comfortable fit were its most important attributes.

**Conclusions:**

The perceived ease of use of MFNS may help providers appropriately treat patients with allergic rhinitis and may improve patient adherence to INS use.

## 

Allergic rhinitis (AR), characterized by nasal congestion, rhinorrhea, sneezing, and itching, is among the most common allergic diseases in the United States, with prevalence estimated between 20 and 40 million people, and is the cause of approximately 14 million physician office visits each year.[1-4] In the United States, AR is associated with an estimated $4.5 billion in direct costs and 3.8 million days of lost work or school.[5-7] In Europe, the annual direct cost of AR is estimated at €1.29 billion.[8] The disease causes significant discomfort and has a substantial impact on patient quality of life, contributing to sleep disorders, fatigue, mood disorders, sexual dysfunction, substantially reduced work performance, and days lost at work.[9-15] School attendance also suffers as a result.[16,17] Intranasal corticosteroids (INS) are recommended as first-line therapy for AR because of their demonstrated efficacy in reducing AR symptoms and favorable safety profile compared with systemic corticosteroids.[18-20]

Patient compliance with a prescribed treatment regimen can significantly affect its ultimate success; this is particularly true of INS, which must be used regularly to be effective. Patient acceptance of, and satisfaction with, the therapeutic regimen are therefore extremely important. Results of recent studies indicate that patients' perceptions of INS are influenced not only by sensory attributes of the agent but also by the ease and comfort of its administration.[21-23] Important features that could affect patient adherence include differences in delivery devices, handling characteristics, and ease of use. Because a positive correlation between patient preference and compliance has been noted, it is important to consider patient acceptability when deciding which INS to prescribe.[14,21,24,25]

Mometasone furoate nasal spray (MFNS) is an INS indicated for the treatment of nasal symptoms associated with seasonal and perennial AR in adults and in children aged 2 years or older. It is also indicated for prophylaxis therapy for seasonal AR in adults and adolescents aged 12 years or older.[26] The objective of the present survey was to determine whether the characteristics of the MFNS delivery device are an impediment to its use.

## Methods

### Subjects

This ease-of-use survey was conducted by Synovate on behalf of Schering Labs, a division of Schering Corporation. A random sample of preidentified MFNS users was recruited from the Synovate interactive national panel through e-mail invitations asking for their participation in the study. Online interviews were conducted in November 2006.

### Assessments

Respondents were asked 11 questions assessing their attitudes about ease of use of MFNS (Table 1). To determine which characteristics were most important to users, respondents were asked to make a more critical assessment of product attributes through a maximal differential technique, based on a Latin square (balanced order and pairing) design. Respondents viewed 5 claims about product attributes at once and were asked to select which were the most important and which were the least important attributes. Each claim was exposed and reacted to 3 times, providing a more robust evaluation than a single assessment. The analysis for each criterion was summarized into 1 measure. The maximal differential analysis does not use a scale; it derives measures without rating the claim.

**Table 1:** Survey Questions About Ease of Use of the MFNS Applicator*

1. Do you find MFNS easy to use?
2. Do you find MFNS easy to store?
3. Would you say MFNS is easy to keep clean/maintain?
4. Would you say MFNS is easy to pump/press?
5. Do you find the mist from MFNS easy to administer?
6. Do you feel that MFNS fits comfortably in the hand?
7. Do you find MFNS comfortable to hold while administering a dose (or mist in the nose)?
8. Would you say MFNS fits well in the hand when administering a dose (or mist in the nose)?
9. Would you say the MFNS applicator fits comfortably in the nostril?
10. Would you say you like using the MFNS pump bottle?
11. Would you say the nozzle is appropriately sized to administer the medicine?

*The survey questions were asked in the order presented here.

## Results

### Subjects

The random sample included 1544 respondents, of whom 506 were pediatric (aged 2-17 years; half were administered MFNS by a caregiver and half self-administered MFNS), 355 were elderly, and 562 (including 227 elderly) were arthritis sufferers (some respondents may have been in more than 1 group). The demographics of the sample population are summarized in Table 2.

**Table 2:** Subject Demographics

	Total Sample (N = 1544)	General MFNS Users (n = 1126)	Pediatric Users (n = 506)*	Elderly Users (n = 355)	Users With Arthritis (n = 562)
Age, yrs					
≤ 18	509 (33)	56 (5)	506 (100)		
18-34	108 (7)	203 (18)			22 (4)
35-44	139 (9)	236 (21)			45 (8)
45-49	139 (9)	191 (17)			67 (12)
50-64	448 (29)	372 (33)		153 (43)	303 (54)
≥ 65	201 (13)	68 (6)		202 (57)	124 (22)
Sex					
Female	988 (64)	709 (63)	253 (50)	266 (75)	438 (78)
Male	309 (20)	304 (27)		89 (25)	124 (22)
No answer	247 (16)	101 (9)	253 (50)		

Values were expressed as no. (%).

*There were 2 subgroups within the pediatric sample of the study. Half of the pediatric patients self-administered MFNS, and half had MFNS administered by a caregiver (ie, parent). Of those administered by a caregiver, 100% were female (mother of the child). For those who self-administered, because of the children's age (< 18 years), sex and other demographic information were not provided.

### Total sample

In the total sample, 98% reported that MFNS is easy to use, 96% reported that the mist from the applicator is easy to administer, and 96%reported that the applicator is easy to pump and press (Table 3). Similar numbers were noted for the questions about the applicator's comfort in the hand. Overall, 85% of total users said that they liked using the MFNS pump bottle.

**Table 3:** Order of Importance of Attributes

			Pediatric Users				
					
Attributes*	Total Sample (N = 1544), %	General Users (n = 1126), %	Total (n = 506), %	Caregiver Administered (n = 253), %	Self-Administered (n = 253), %	Elderly Users (n = 355), %	Users With Arthritis (n = 562), %
MFNS is easy to store (Q2)	98	98	98	98	98	99	98
MFNS is easy to use (Q1)	98	98	98	99	98	97	97
MFNS is easy to keep clean/maintain (Q3)	97	97	96	97	95	96	97
MFNS applicator fits comfortably in the nostril (Q9)	96	97	94	92	96	97	98
MFNS is easy to pump/press (Q4)	96	96	96	95	96	95	95
The mist from MFNS is easy to administer (Q5)	96	96	96	97	95	95	95
MFNS fits comfortably in the hand (Q6)	96	96	97	97	96	97	96
The nozzle is appropriately sized to administer the medicine (Q11)	95	96	93	91	94	97	96
MFNS is comfortable to hold while administering a dose (or mist in the nose) (Q7)	94	94	94	95	93	95	93
MFNS fits well in the hand when administering a dose (or mist in the nose) (Q8)	94	94	95	96	93	95	93
I like using the MFNS pump bottle (Q10)	85	85	85	93	77	84	83

Values were presented as the percentage of users with positive answers to questions.

*The attributes were listed by general order of derived importance rather than by question number.

### Pediatric users

Among pediatric users, 96% of those who administer MFNS themselves said that the "applicator fits comfortably in the nostril"; 92% of caregivers agreed with that statement. Nearly 100% of those in both groups reported that MFNS is easy to use, and more than 90% said that it was easy to administer and that the applicator was comfortable to hold. However, although 93% of caregivers reported that they liked using the MFNS pump bottle, only 77% of self-users agreed with that statement, the lowest percentage of any of the subgroups.

### Elderly users and users with arthritis

Nearly all elderly users and users with arthritis agreed that the "applicator fits comfortably in the nostril" (96% and 97%, respectively) and is "appropriately sized to administer the medicine" (97% and 96%, respectively). These rates were similar to those of the general users. Both subgroups found MFNS easy to administer (≥ 95%) and to pump/press (95%). Ninety-five percent of elderly users and 93% of users with arthritis reported that the applicator was comfortable to hold and fit well in the hand. The response rate to "like using the MFNS pump bottle" was 84% for elderly users and 83% for users with arthritis.

### Maximum differential analysis

All categories of MFNS users selected ease of administration features such as "the mist is easy to administer," "is easy to use," and "is easy to pump/press" as the most important attributes (Table 4). Other characteristics considered important among pediatric caregivers, the elderly, and users with arthritis included "the applicator fits comfortably in the nostril" and "is appropriately sized to administer the medicine." The least important characteristic was "is easy to store."

**Table 4:** Derived Importance of Ease-of-Use Survey Statements*

		Pediatric Users				
				
Base: Total (n)	General Users (n = 1126)	Total (n = 506)	Caregivers (n = 253)	Self-Administered (n = 253)	Elderly Users (n = 355)	Users With Arthritis (n = 562)
The mist from MFNS is easy to administer	177	179	178	181	178	175
MFNS is easy to use	168	170	164	176	170	166
MFNS is easy to pump/press	157	160	154	167	158	156
The MFNS applicator fits comfortably in the nostril	144	150	154	145	144	143
The nozzle is appropriately sized to administer the medicine	128	135	144	126	126	126
MFNS is comfortable to hold while administering a dose (or mist in the nose)	95	84	81	88	93	97
MFNS fits well in the hand when administering a dose (or mist in the nose)	81	73	72	74	81	85
MFNS is easy to keep clean/maintain	79	82	92	72	78	79
MFNS fits comfortably in the hand	42	38	36	40	42	45
I like using the MFNS pump bottle	42	38	38	39	41	41
MFNS is easy to store	15	14	16	12	14	16

*The ranking data in this table were derived from patients' selection of most important survey statements, with the larger numbers identifying the most important; the methodology used was a maximum differential analysis.

## Discussion

Individual product attributes of different INS are readily distinguishable by patients. Patient acceptance and satisfaction with these attributes may influence their medication preferences and, ultimately, treatment adherence. One survey conducted in 2000 adults with AR found that 80% of respondents were noncompliant with a range of treatments for various reasons, including that the medication was inconvenient to take.[27] A lack of adherence to INS therapy can then lead to inadequate treatment of AR and subsequent problems such as a greater number of office visits and decreased productivity and increased absenteeism at work or school.[28] In light of these facts, the results of this survey, showing that the great majority of patients using the MFNS applicator agree that it is easy to use, are important.

This survey reports data for subgroups of INS users: pediatric patients, elderly patients, and individuals with arthritis. The results support those of a recent product accessibility study conducted on behalf of the Arthritis Foundation, where MFNS was evaluated by 8 subjects with arthritis (mean age, 65 years) to determine accessibility and ease of use. Ten tasks were administered: 6 tasks were related to opening the package and reading information and 4 tasks were related to physical use. Subjects rated MFNS on a scale ranging from 1 = not at all easy to use to 5 = extremely easy to use. The mean overall score for all 10 tasks was approximately 4.25, indicating a high degree of ease of use of MFNS among patients with arthritis.

## Conclusions

Among all categories of MFNS users in this survey, greater than or equal to 97% reported that MFNS is easy to use. Nearly all pediatric users, elderly users, and users with arthritis agreed that the applicator fits comfortably in the nostril and is appropriately sized to administer the medicine, and that the drug is easy to administer. The high ratings given to the ease of use of MFNS in this survey may support providers in their efforts to improve patients' adherence to AR treatment regimens.

## Note

Dr Fromer has participated in speakers' bureaus for AstraZeneca Pharmaceuticals LP, GlaxoSmithKline, Merck and Co, Inc, Phadia US, and Schering-Plough Corporation, and has served on advisory boards for Boehringer-Ingelheim Corporation and sanofi-aventis US LLC. Mr Ortiz has served as consultant for ALTANA Pharma, AstraZeneca Pharmaceuticals LP, Genentech, Inc, Novartis AG, Sanofi-Aventis US LLC, Schering-Plough Corporation, and TEVA Pharmaceuticals USA, and has sat on speakers' bureaus for Abbott Laboratories, Alcon Laboratories, Inc, AstraZeneca Pharmaceuticals LP, Genentech, Inc, Novartis AG, Pfizer Inc, sanofi-aventis US LLC, Schering-Plough Corporation, and TEVA Pharmaceuticals. Ms Dowdee does not have any financial interest related to this work.
